# The Law of Rape—Chloroform in Rape Cases

**Published:** 1860-06

**Authors:** M. B. Walker, J. C. Reeve

**Affiliations:** Attorney for Defendant, Dayton, Ohio; Dayton, Ohio


*The Law of Rape—Chloroform in Rape Cases.* Reported by M. B. Walker, Esq., Attorney for Defendant, with Remarks by J. C. Reeve, M. D., both of Dayton, Ohio.

[Court of Common Pleas of Mercer County, Ohio, January Term, 1860.]

THE STATE OF OHIO *vs.* DAVIS GREEN.

The evidence in this case tends to show that Jane Gray, the prosecutrix, is a truthful, virtuous girl, robust and healthy, of limited education and intelligence, though of good natural sense, aged seventeen years, on 21st August, 1857; that on the night of the 23d June, 1857, she lodged in a bed with a daughter of defendant about of same age, in the northeast corner room of a village hotel in Mercer county; that in the adjoining room south, there lodged a man and his wife, and in the adjoining room west, with an unfastened door between, there lodged the defendant and other persons in other beds; that the prosecutrix and her bed companion retired about ten o'clock P. M., and, after talking a short time, fell asleep; that during the night, the first thing remembered by the prosecutrix was, that the defendant had her by the arms pulling her out of bed; that he said to her he was Dr. Green, and that he had come to have sexual intercourse with her; that he placed her in a position with her feet touching the floor and her weight partially resting on the bed and pillows, and that in that position he had complete sexual intercourse with her; that she experienced the pain of rupture of the hymen, but experienced upon her clitoris a pleasurable sensation from the coition; the act lasted but a few minutes, and upon leaving her the defendant said to her she must never tell it; that it would not hurt her; he held his hand upon her mouth, and she felt a rag between his hand and her mouth; she heard what he said, was conscious of all that occurred, she tried to speak, but left so weak or scared, that she could not, or could not speak loud, and did not say but a word or two—said "Go away—Oh dear!" she tried to force him away, but could not; she experienced a ringing sensation in the head, felt weak, drowsy and sleepy, but did not sleep any more that night; she remained in bed until morning, made no outcry, and told no one of the occurrence until about last December, 1857; next morning she felt unwell and presented a sad and gloomy countenance, and for a week or two was nervous and easily alarmed; the ringing in the head lasted a day or two; for three or four days she could not sit up for any considerable time; the symptoms of weakness lasted two weeks. That time, 23d June, was the usual period for the return of the menstrual discharge, and symptoms of it were felt, but no actual discharge had yet occurred. On the morning of the 24th she observed a spot like blood on her chemise, the only night-dress she wore, which she supposed was a slight menstrual discharge, but that no discharge followed at any time thereafter. She conceived and gave birth to a child on the 26th March, 1858. After retiring to her room on night of the 23d June, before going to bed, her nose bled. She never saw chloroform before, but smelled it on trial and believes the smell to be like that she experienced on night of 23d June. She first thought defendant had intercourse with her twice that night, and had told others so, but on reflection, was sure that it was only once; she saw him with shirt and drawers on, but no other clothing; she made an effort twice, with both hands, to resist him, but could do nothing. She weighed one hundred and thirty pounds, was in good health and had always enjoyed good health. Did not smell medicine when first awoke, but did after defendant left her room, in about six mimutes; the effect was unpleasant—cannot say painful; her mind was clear from the time she awoke, and she knew everything. Her feet were about six inches apart—more than half her weight on her feet, the rest thrown back on the upper part of the bed; the rail of the bedstead came in contact with the middle of her thighs She made no effort to awaken the daughter of the defendant, though her head was near or touching her; did not halloo or call anybody; her hands were not restrained at any time. Defendant only touched her with one of his hands; is sure that she remembers everything that occurred accurately.

The defendant is a physician. There was a large amount of testimony on both side, tending to prove the charge and tending to disprove it. The daughter of defendant, a highly intelligent young lady, swears that she slept on the front side of the bed, was not disturbed in the night and smelled no odor of medicine of any kind; saw nothing unusual in the appearance of prosecutrix next morning. The defendant was just recovering from a long and severe attack of phlegmonous erysipelas, the left hand very sore, and poulticed, the neck very stiff and sore, and the right hand also sore and in ulcers. No one about the house heard any noise or disturbance during the night, after parties had retired. The partitions between the rooms were of boards, had shrunk so that there were cracks between the boards one half inch in width—boards were one inch in thickness—had stood for twenty years; the bed was of ordinary size.

N. L. Hibberd, J. S. Conklin, J. H. Hart and Joseph Plunket, for State.

M. B. Walker, F. C. Le Blond, E. M. Phelys, C. P. Edson and P. Depuy, for Defence.

M. B. Walker, for defence, cited the following authorities: Dr. Snow on Anæsthetics, pp. 34 to 48, inclusive; *Ib.*, 98; Wharton & Stille's Med. Jurisp., Secs. 728 to 733, inclusive, not *j; Ib.*, Sec. 443, note *q;* Dunglison's Physiology, Vol. II., pp. 368, 450, 420, 423, 470, 369, 423, 465, 424, 425 and 427; Wilson's Anatomy, pp. 548, 550, 361, 366.

The evidence and arguments of counsel being closed, the Court, Wm. Lawrence, judge, charged the jury, substantially, as follows:

The indictment charges the defendant with rape upon Jane Gray, on the 23d June, 1857. The defendant pleads not guily. This issue thus made you have been sworn well and truly to try. The indictment is drawn upon the 5th Secton of the Crimes' Act of March 7th 1835 (Swan's R. S., 269, 1 Crimin. 184) which provides, "that if any person shall have carnal knowledge of any other woman or female child than his daughter or sister, forcibly and against her will, every person so offending shall be deemed guilty of a rape," etc.

In this case rape may, therefore, be defined the unlawful carnal knowledge of a woman other than the daughter or sister of the accused, forcibly and against her will. The definition is only important as it may serve to call the attention of the jury to the facts which must be provided, in order to warrant a verdict of guilty, which are these: That Jane Gray was not the daughter or sister of the defendant (Crimes, Sec. 5 ); that he had carnal knowledge of her, which requires penetration and emission (Williams *vs.* Ohio, 14th Ohio R. 222); that it was had forcibly; that it was against her will; that it was thus had in Mercer Co., Ohio. These facts must be established by lawful evidence (the Judge then stated the law—the province of the jury to determine the facts; that of the court to determine the law; the presumption of innocence; the law as to reasonable doubts, etc., substantially as in Rollins *vs.* Ohio, 8th Ohio St. R. 148, 152; and after explaining sufficiently the several facts to be proved, proceeded as follows): The carnal knowledge must also have been *against the will of Jane Gray.* If unlawful sexual intercourse should be proved, the law does not *presume*, from that fact alone, that it was against the will of the female; there must be sufficient evidence, by circumstances or otherwise, of that fact. (See State *vs* Crow, 10th West. Law Journal, 501.) But if the facts and circumstances show that the defendant forcibly had carnal knowledge of Jane Gray, without her consent *to the act*, he knowing that fact, that is sufficient evidence that it was against her will. (See Wharton's Crim. Law, Secs. 1141, 1146, n.; Wharton & Stille's Med. Jur., Secs. 459, 463; Regina *vs.* Camplin, 1 Car. & K., 756; 1 Denis' C. C., 90. If the defendant forcibly had unlawful sexual intercourse with Jane Gray with out her consent, and the act was commenced and completed by penetration and emission while she was asleep, unable to know the fact, or before she was sufficiently awake to enable her will and understanding to determine the nature and consequences of the act, and the defendant knew these facts, then he is guilty of rape. Wharton & S.'s Med Jur., Secs. 440, 443, n. *q;* Wharton's Crim. Law, Secs. 1146, 297, 631, 2159; 1 C. & K., 746; 1 West. Law Monthly, 333; 3 Gr. Ev., Sec. 211; 14 Ohio Rep., 222.

There is, perhaps, but a single published case of alleged rape effected by means of chloroform—that of Dr. Beale, of Philadelphia, published in Wharton & Stille's Medical Jurisp. The defendant was pardoned by the Governor of Pennsylvania. The subject of chloroform is very fully discussed in a late valuable work by Dr. Snow, of London, on Anæsthetics.

If the defendant administered chloroform to Jane Gray, and thereby rendered her unconscious—without will—and in that condition the defendant, knowing it, had carnal knowledge of her forcibly, then he is guilty of rape. Wharthon & Stille's Med. Jur. Secs. 458, 459.

Generally, the crime of rape is committed upon females in the enjoyment of all their faculties. In such cases the inquiry, whether the crime was against the will of the female, is determined by evidence of acquiescence or resistance; and as the State is required to prove the *absence of consent*, in order to make out guilty, a prosecution will generally be defeated by evidence of *acquiescence*. This must always be so when the prosecutrix is sufficiently in the enjoyment of her faculties to understand the nature and judge of the consequences of sexual intercourse, or when the defect of capacity, which induces acquiescence, is unknown to the accused. If the prosecutrix, having the capacity to understand the nature and judge of the consequences of sexual intercourse, and the power to resist by act or word, and neither such capacity nor power was overcome by force, fear, or chloroform, her acquiescence in the act would defeat a prosecution for rape. In such case, any consent thus given, however reluctantly, even if the judgement and conscience did not approve the act, but if the will yielded to the influence of sexual desire or otherwise motive, there can be no rape. In such case, passive submission is evidence of acquiescence; and if her conduct was such that the defendant might fairly infer that she acquiesced, and he did so infer, then he is not guilty of rape. If she acquiesced through force, fear, chloroform or any defect of capacity, but the cause of such acquiescence was unknown to defendant, he is not guilty of rape. 1 West. Law Monthly, 333. But the mind is composed of various faculties or powers, each operating and affecting others more or less remotely. The destruction or suppression of one may defeat or pervert the capacity of another, and thus the power to acquire just perceptions or form just conclusions upon some, or all subjects, may be impaired or annihilated. This idea is illustrated in a note to Sec. 443 of Wharton & Stille's Med. Jur., in relation to certain experiments with chloroform, where it is said: "In the above observations it may very plainly be seen that the will no longer exercises its control over the mental operations. The thoughts run headlong upon their accustomed track, or in any direction in which they may have been impelled by fortuitous impressions made upon the nerves of general or special sensation; there is no power to restrain them, and, while the dream is a pleasant one, no desire to do so." It is the right of every human being to enjoy all these faculties in the fulness of their natural vigor. Webster has defind the *will* to be "that faculty of the mind by which we determine either to do or forbear an action; the faculty which is exercised in deciding, among two or more objects, which we shall embrace or pursue;" and he adds, "the *will* is directed or influenced by the *judgment*, the understanding or *reason* compares different objects which operate as motives; the *judgment* determines which is preferable, and the *will* decides which to pursue. In other words, we *reason* with respect to the value or importance of things; we then *judge* which is preferred, and we *will* to take the most valuable. These are but different operations of the mind, soul, or intellectual part of man." I present these metapysical views (whether correct or not the jury will determine) merely to illustrate the principle of law I am about to state. When the *will* acquiesces in *coition*, there can not, as a general rule, be any rape; but the acquiescence which defeats a prosecution for rape, is that of a will so far under the enlightened guidance and control of the other faculties, that the mind can fairly comprehend the nature, and judge of the consequences of the act, unless the defect in capacity is unknown to the accused. If the faculties have been to some extent suspended by chloroform, but enough remain to reasonably comprehend the nature and judge the consequences of the act, then acquiescence in coition will defeat a prosecution for rape. But if through the influence of chloroform, either directly upon the *will* or the *consciousness*, or the *faculties* of the mind, or the sexual feelings and emotions, (see Wharton & Stille's Med. Jur., Sec. 443, note,) the mental capacity is so benumbed, suspended or perverted as to be unable reasonably to comprehend the nature and judge the consequences of coition, and by reason of such condition known to the defendant the act is acquiesced in or consented to, such acquiescence or consent will not alone defeat a prosecution for rape; rape may exist with acquiescence thus knowingly obtained.

It is of the utmost importance that you should ascertain whether chloroform was administered, and if so, whether it deprived the prosecutrix of mental and physical powers. (The Judge then called the attention of the jury to the evidence tending to prove and disprove the administration of chloroform.) The jury will find it important to ascertain whether chloroform could be administered during sleep; whether, if attempted, it produced the waking state, and, if administered, its effect upon *consciousness*, the *muscular power*, the organ of *speech*, the *memory* the *will* faculties, *sexual* excitement, the capacity of *conception*, its tendency to produce *anæthesia* and *delusion*, and these in all the various stages of its effect upon the *mind* and *body*, both in passing into, during, and in passing out of its effects.

If it be assumed (and whether it should be is for the jury to say) that there is evidence tending to show that chloroform was administered to the prosecutrix while asleep; that sexual intercourse was had with her; that she partially or wholly awoke before it commenced; that she was conscious of it and all the movements attending it; that she could and did hear and understood words spoken in a low tone; that the intercourse produced upon her clitoris a pleasurable sensation; that this was preceded by the pain of a ruptured hymen; that she did not speak; that she felt a desire to resist physically, endeavored to do so, but could not; that the act was followed by pregnancy, and the birth of a child, in two hundred and seventy-six days; that she was a vigorous girl, in her seventeeth year, virtuous, truthful, of limited education and intelligence; that the act was at the proper time for the return of the menstrual period, but before any actual discharge, it will be important to ascertain whether there is any stage, in the effect of chloroform upon the human system, when these facts can exist consistently with the idea that such intercourse could be had without her consent.

In the case of Brown *vs.* Jamison, in the Superior Court of Cincinnati, January 18th 1860, on motion to discharge an attachment, the defendant having alleged that chloroform was administered to her while asleep, whereby she was robbed, Judge Storer remarked: "In relation to the administration of chloroform to the defendant and her daughter, the court had considered this branch of the case with the utmost deliberation. The evidence of the physicians was to the effect that they had never known of a case in which a person was placed under the influence of chloroform without being woke up, and never heard of any, except in newspaper reports, and that the influence lasted only from five to fifteen minutes, unless where the application was repeated; whereas, in this case, if the operation had commenced at two o'clock in the morning, the daughter of this lady must have been under the induence six hours, and defendant herself about twelve hours. Other narcotics would have produced these results, and there was no evidence that chloroform had been used, except from the fact that it was found permeating the atmosphere. The court were led to the conclusion, and it afforded as much pain as the contrary result (could they conscientiously have arrived at it) would have afforded pleasure, that there was no sufficient evidence that the robbery was committed; they should, therefore, set the attachment aside." But the Cincinnatti *Gazette* of January 21st, referring to the same case, thus speaks:

"*Interesting Experiments with Chloroform.*—In a robbery case tried before the Superior Court this week, in relation to the administration of chloroform to defendant and her daughter, physicians testifid that they had never known of a case in which a person was placed under the influence of chloroform without being woke up. With this statement in view, we understand that Dr. Miller administered chloroform to ten (10) of the inmates of the Commercial Hospital, eight of whom remained under the influence without waking, while the remaining two confirmed the testimony of the physicians before the court."

The medical authorities show that the human female is very susceptible to impregnation for a day, at least, preceding the menstrual discharge; then she is less so during the discharge, which usually continues about four days, because the male semen is liable to be carried off by it; that she is again, after it, very liable to conception until the ovum is expelled from the uterus and vagina. The termination of every menstrual period is followed by the discharge of an ovum generally in five or six days, which may be detected in the form of a greenish or grayish, tough mucous globule, about the size of a pea, either on water or by wearing a bandage. This is generally preceded by a slight watery discharge and parturient pains, barely perceptible. When the ovum has passed away, impregnation is impossible until the recurrence of the sensations preceding the menstrual flow, unless an ovule is detached from the ovaries by some irregularity of nature or violence. This proceeds on the idea that fecundation occurs by the contact of the female ovum with the male semen; injected into the female organs of generation during the day preceding the menstrual flow, is retained, and impregnates the ovum afterwards detached, and that the ovum, in its passage from the ovaries through the Fallopian tubes and the uterus, may be impregnated at any time before it is finally discharged. This inquiry may be assisted by ascertaining whether the various powers of the mind and body fade away, under the influence of chloroform, gradually and co-equally, and return in like manner, as the influence passes off, or whether some, and if so, what ones precede in thus fading away and being restored, and the order thereof, in all the various stages of the influence, and whether some, and if so, what faculties are retained, and the extent and capacity of them. In the case which I have assumed, where the sense of hearing remained, and the sensations of pain and pleasure were felt in a greater or less degree, these facts would tend to show that the stage or condition of *anœsthesia* had either not been reached or was passed; and if so, it might be much more probable that memory would retain its power than if the facts ran otherwise; and if the capacity to remember existed, statements made by its aid might be reliable. But as failure to resist by *word* and *act*, having the capacity to do so, would be strong, if not sufficient evidence of acquiescence in the coition, it would at once become necessary to determine if the faculties of hearing and feeling could co-exist in a sound body without either the capacity to speak or make forcible resistance. If that be not possible, then due weight should be given to such consideration in determining whether she acquiesced in the coition. But if the capacity to hear, feel and remember be consistent with incapacity to speak or forcibly to resist, then the evidence of guilt may thereby be enhanced. What may be the truth, you will determine from the evidence in the case. But if the prosecutrix had the capacity to hear, feel and remember, and a capacity to speak and forcibly resist, but the inclination to do so was lost—the will overcome by the action of chloroform either operating upon the will-faculty or the *judgment* and *reflective* faculties, (or sexual emotions,) so that the mind was thereby incapable of fairly comprehending the nature and consequences of sexual intercourse—and the defendant, knowing these facts, had unlawful carnal knowledge of her forcibly, that would be a rape; and it would be, in such case, wholly immaterial whether the entire mind was disordered and overthrown, or only such faculties thereof as rendered it incapable of having just conceptions and drawing therefrom correct conclusions in relation to the alleged rape. Whether the physical and other mental capacities I have named could operate normally, while faculties of the mind, as the judgment, the understanding, the reflection and reasoning faculties were so deranged or overthrown as to destroy the capacity to comprehend the nature and consequences of coition, is a question of fact for the jury to determine upon all the evidence in the case. But if the prosecutrix had the capacity to hear, feel, remember, to speak and resist, or in any event, it should not be presumed that her will was overcome without proof of the fact beyond a reasonable doubt. If chloroform may produce *delusion* in the mind of its subject in any of its stages, you will inquire if it existed in this case—whether its existence is consistent with the other mental and physical phenomena which you may find to have existed; and you will give due effect to your conclusions upon this subject.

With these principles, as to what facts are necessary to constitute rape, the jury will proceed to inquire into the prominent points of controversy, and ascertain if it is proved that the defendant forcibly had unlawful carnal knowledge of Jane Gray, and if so, was it against her will.

[The Judge then read to the jury Section 212 of 3d Greenl. Ev., and Section 468 of Wharton & Stille's Med. Jur., and called the attention of the jury to the prominent points of evidence relied upon to prove and disprove the fact of sexual intercourse, and upon the subject of acquiescence.]

Verdict of the jury, *Guilty;* motion for new trial overruled.

Motion in arrest of judgment continued to next term, by agreement of counsel.

---

*Remarks.*—The interest which attaches to every case of crime attempted or committed by means of anæsthetic agents demands some remarks upon the medico-legal points in the above trial.

So far as we are aware, but one case has been reported of the commission of rape while the female was under the influence of chloroform. We allude to the celebrated Beale case of Philadelphia; in that, however, there was no doubt in regard to the inhalation of the chloroform; it was given for a proper purpose at a proper time and place, and the question was whether the prosecutrix really suffered a violation of her chastity or was deceived by an erotic dream, which anæsthetics are now well known to produce, the impression of which, after awakening, had all the vividness of reality. In the present case, on the contrary, there was no call or excuse for inhalation of chloroform; if given, its administration must be looked upon as a part of the crime itself.

There is an important point, however, in which the two cases are similar, and one which we have not yet seen examined in any work on medical jurisprudence. The prosecutrix in each case distinctly swears that she was conscious of what occurred at the time the offence was committed, and that she suffered pain, yet that she was at the same time deprived of the power of making any resistance or outcry. The question, therefore, for the medical jurist to answer is—Does chloroform produce a condition of the nervous system in which consciousness and sensation are perfect, but volition is abolished? This question did not assume the first importance in the Beale case, while in the one under consideration it must be looked upon as the vital point in the medico-legal aspect of the case.

We do not believe that any one who has had experience in the administration of chloroform would hesitate in giving a negative reply to this question. When this agent is inhaled, it produces its effects in a *gradual* manner; certain portions of the nervous system submit to its influence before others, and the order is well marked, the functions of the cerebrum being first abolished, then those of the cerebellum, afterwards those of the spinal system. Every person who has watched its effects knows that consciousness is deranged and lost before the stage of excitement occurs—a stage in which there is often a great deal of muscular exertion, such as struggling, and trying to rise from the couch or chair, with loud talking, and that this stage must be passed before the sensibility of the patient is sufficiently abolished for the performance of a surgical operation. From what we know of the effects of chloroform, we do not hesitate to say that there is no such thing as a patient being deprived by it of the power of speech and of voluntary motion without sensation and consciousness being also abolished.

Our opinion is supported by the authority of all writers upon the subject. Snow, in his work on Anæsthetics, enters very fully into a consideration of the physiological effects of chloroform;* as several pages are taken up with this description, we cannot copy it here, but present a brief abstract of the different "degrees" of the influence, as he gives them:

In the *first degree* he includes "all the effects of chloroform that exist while the patient retains a perfect consciousness of where he is and what is occurring around him;" in this degree "there is often a considerable diminution of common sensibility" —"in a few cases, the abstraction of a tooth and other minor operations have been performed without pain, whilst consciousness has been retained."

"In the *second degree* of narcotism there is no longer correct consciousness. The mental functions are impaired, but not necessarily suspended."—"There is generally a considerable amount of anæsthesia connected with this degree of narcotism. . . . . . Loss of sensation is indeed sometimes so complete in this degree, especially in children, that the surgeon's knife may be used without pain. . . . . Although the patient is generally silent, he may, nevertheless, laugh, talk, or sing. . . . . He feels the inconvenience of the vapor he is inhaling, . . . . and endeavors to push away the inhaler."

In the *third degree* "there are no longer any voluntary emotions," rigidity and spasms of the muscles occur—the patient mutters in an almost inarticulate and a perfectly unintelligible manner," but "is quite incapable of any perception or consciousness of pain."

In the *fourth degree* the breathing is stertorous, the pupils dilated, the muscles completely relaxed, and the patient perfectly insensible.

In the *fifth degree* the respiration becomes difficult, feeble or irregular, and finally ceases, if the inhalation be continued, and is followed by cessation of the heart's action and death.

---

*On Chloroform and Other Anæsthetics; their Action and Administration. By John Snow, M. D., London, 1858: pages 34–43.

Essentially the same description of the gradual influence of chloroform, and of the order in which the different parts of the nervous spstem are affected, is given by Druitt in the *Surgeon's Vade Mecum*;* he says: "It [chloroform] begins by affecting the mind and consciousness. In its smallest dose it stimulates, then disturbs, then suspends the mental operations. It next diminishes the power of the nerves in receiving and communicating, and of the brain in perceiving sensations, whether arising from causes within the body or without; hence it diminishes or abolishes the perception of pain."

Erichsen, in his *Science and Art of Surgery*,† says: "The first influence of chloroform appears to be exercised upon the nervous system. The patient becomes excited and talkative, and a state of unconsciousness is induced. . . . . As the administration of the chloroform continues, however, complete paralysis of sense and motion is induced."

We can adduce far more numerous descriptions of the manner in which ether produces its effects than of chloroform, and if it be objected that this was not the agent employed, and cannot, therefore, bear upon the case, it can be shown that ether and chloroform affect the nervous system in precisely the same order. Snow makes this statement repeatedly, and quotes from Flourens‡ a brief description of the action of ether, which, he says, "will apply equally well to chloroform;" in this description the regular succession and order in which the nervous centres lose their powers is so distinctly and briefly stated that we quote it entire: "First, the cerebral lobes lose theirs, viz., the intellect; next, the cerebellum loses its, viz., the power of regulating locomotion; thirdly, the spinal marrow loses the principle of sensitiveness and of motion; the medulla oblongata still retains its functions, and the animal still continues to live: with loss of power in the medulla oblongata, life is lost."

Again: M. Gerdy tried the effect of ether upon himself, "with the object of observing closely its successive phenomena, and found that, with the exception of the vibratory and benumbed

---

*Seventh Edition: London, 1856: page 707.

†American, from the Second London Edition, page 31.

‡On page 45 of his work from Gaz. des Hospitaux, 20 Mars, 1847.

sensation which rendered the sense of touch and of pain obtuse, and the noise in the ears which dulled the sense of hearing, his intelligence was clear, his attention active, and his *will* so firm that he willed to walk, and did walk, in order to observe the effect upon his locomotion."*

It will be remarked that all agree upon the fact that unconsciousness is induced before sensation and motion are abolished. Let us turn to writers upon medical jurisprudence, to see what they say upon the subject. We quote first from Wharton and Stille, section 442, note *q:*

"That advanced stage of etherization in which perfect narcotism is produced, is, in reference to the present question, of considerable importance; for if the power of resistance is then lost, so also is the consciousness of a real motive for it. To be more explicit, if an outrage be perpetrated upon a woman lying wholly helpless and unconscious, she cannot be aware of the liberties which are being taken with her person, and will not, therefore, make any opposition to them. She cannot, moreover, afterwards describe, with elaborate detail, the manner and particulars of the assault, and yet have been incapable of withdrawing from or repelling it. If her muscles and voice have been paralyzed, so also has her outward consciousness. Voluntary muscular movement is not paralyzed until the state of narcotism is produced, at which time, however, all outward consciousness is extinct."

Without entering into a full consideration of the effects of anæsthetic agents, the following propositions as to the different degrees of their influence, are given in the last edition of Beck's *Medical Jurisprudence.*†

*First.* A state of insensibility may be induced, rendering the person as completely unconscious of the violation of her chastity at the time as if she was fully narcotized by opium or any stupefying drug.

*Second*, She may be rendered partially unconscious, or thrown into a state in which she has no adequate appreciation of the outrage, although more or less cognizant to its committal.

*Third*, The power of opposition, either by words or actions,

---

*Wharton and Stille's Medical Jurisprudence: quoted from Bulletin de l'Academie, vol. xxi., page 304.

†Eleventh Edition, vol. i., page 246: note by Austin Flint, M. D.

may be taken away or impaired, even if the faculties of the mind are retained sufficiently to understand the intention of the criminal party.

It may seem at first, that because this last proposition favors the existence of a state in which the power of resistance is abolished, and yet the patient is aware of what occurs around her, it can be used in making out the case of the prosecutrix. When anæsthetics were first introduced into practice we often read of cases where the patient watched, smiling, the different steps of an operation, or talked cheerfully during its progress. Such cases we have read of, but have never seen; nor are they common, for Snow saw but one in the whole course of his experience, which amounted to the administration of chloroform over four thousand times and of ether nearly two hundred times; once he saw a child hold a toy in its hand and look at it attentively while being cut for stone. There is one very important fact to be considered by those who would apply this proposition and these cases to the case before us: it is, that the one says nothing of *sensation* remaining as well as consciousness, and in the other we know that it is abolished from the absence of all expression of suffering, without which the administration of the anæsthetic agent would have been a failure. The prosecutrix in this case not only swears as to events that occurred and words which were spoken, but swears positively that she suffered pain and experienced pleasure. As there is not a particle of evidence that the will can not be exercised so long as sensation remains, although occasionally it may happen that volition is abolished while consciousness of external events remains, we cannot believe that the prosecutrix was deprived by chloroform of the power of making outcry or resistance.—*Cincinnati Lancet and Observer.*

